# Lack of a protective effect of the *Tmem106b* “protective SNP” in the *Grn* knockout mouse model for frontotemporal lobar degeneration

**DOI:** 10.1186/s40478-023-01510-3

**Published:** 2023-01-27

**Authors:** Anne-Sophie Cabron, Uwe Borgmeyer, Julia Richter, Helga Peisker, Katharina Gutbrod, Peter Dörmann, Anja Capell, Markus Damme

**Affiliations:** 1 Institute of Biochemistry, Christian-Albrechts-University Kiel, Olshausenstr. 40, 24098 Kiel, Germany; 2 Center for Molecular Neurobiology (ZMNH), University Medical Center Hamburg-Eppendorf, Hamburg, Germany; 3 Department of Pathology, Hematopathology Section, University Hospital Schleswig-Holstein, Kiel, Germany; 4 Institute of Molecular Physiology and Biotechnology of Plants (IMBIO), University of Bonn, Bonn, Germany; 5 Division of Metabolic Biochemistry, Biomedical Center (BMC), Faculty of Medicine, Ludwig-Maximilians-Universität München, 81377 Munich, Germany

**Keywords:** TMEM106B, Progranulin, FTLD, Protective SNP

## Abstract

Genetic variants in *TMEM106B* are a common risk factor for frontotemporal lobar degeneration and the most important modifier of disease risk in patients with progranulin (*GRN*) mutations (FTLD-*GRN*). *TMEM106B* is encoding a lysosomal transmembrane protein of unknown molecular function. How it mediates its disease-modifying function remains enigmatic. Several *TMEM106B* single nucleotide polymorphisms (SNPs) are significantly associated with disease risk in FTLD-GRN carriers, of which all except one are within intronic sequences of *TMEM106B*. Of note, the non-coding SNPs are in high linkage disequilibrium with the coding SNP rs3173615 located in exon six of *TMEM106B,* resulting in a threonine to serine change at amino acid 185 in the minor allele, which is protective in FTLD-*GRN* carriers. To investigate the functional consequences of this variant in vivo, we generated and characterized a knockin mouse model harboring the *Tmem106b*^*T186S*^ variant. We analyzed the effect of this protective variant on FTLD pathology by crossing *Tmem106b*^*T186S*^ mice with *Grn*^*−/−*^ knockout mice, a model for *GRN*-mediated FTLD. We did not observe the amelioration of any of the investigated *Grn*^*−/−*^ knockout phenotypes, including transcriptomic changes, lipid alterations, or microgliosis in *Tmem106b*^*T186S/T186S*^ × *Grn*^*−/−*^ mice, indicating that the *Tmem106b*^*T186S*^ variant is not protective in the *Grn*^*−/−*^ knockout mouse model. These data suggest that effects of the associated SNPs not directly linked to the amino acid exchange in TMEM106B are critical for the modifying effect.

**Supplementary Information:**

The online version contains supplementary material available at 10.1186/s40478-023-01510-3.

## Introduction

Genetic variants in *TMEM106B* are a common risk factor for frontotemporal lobar degeneration (FTLD) and the most important modifier of disease risk in patients with progranulin (*GRN*) mutations (FTLD-*GRN*) identified as early as 2010 [43]. Later, *TMEM106B* variants were shown to modulate disease risk and severity in other neurodegenerative diseases, including Alzheimer’s disease, Parkinson’s disease, and chronic traumatic encephalopathy [5, 6, 26, 42]. Besides these genetic associations/ risk alleles, a dominant mutation (D252N) in *TMEM106B* causes hypomyelinating leukodystrophy in rare cases [38]. Very recently, it was shown that TMEM106B could form fibrils upon proteolytic cleavage and aggregation of the luminal domain, and it was speculated that those TMEM106B-fibrils are the major component of protein aggregates rather than TDP-43 in FTLD patients [8, 20, 36].

Genome-wide association studies (GWAS) lead to the identification of several SNPs in *TMEM106B* that are associated with the occurrence of FTLD-TDP [43]. The minor allele of each significant *TMEM106B* SNP was underrepresented in FTLD-TDP patients. These genetic associations are highly robust and reproducible and were replicated several times in independent studies and FTLD cohorts [12, 14, 24, 44]. The most significant association of the *TMEM106B* SNPs is observed in FTLD patients carrying heterozygous loss-of-function mutations in *GRN*, a genetic condition that leads to very high penetrance to FTLD [43]. Interestingly, subjects with the minor alleles of the *TMEM106B* SNPs seem to have a lower probability of developing the disease, thus leading to the assumption that the minor alleles of the associated *TMEM106B* SNPs are “protective SNPs”. While most of the *TMEM106B* SNPs that reach genome-wide significance, including the one with the most significant association (rs1990622), are intronic variants, one nonsynonymous variant (rs3173615) is located in the coding region of exon six of *TMEM106B* [25, 30]. Notably, the other non-coding SNPs are in high linkage disequilibrium with the nonsynonymous SNP rs3173615 [44]. Because the minor allele rs3173615 confers a lower disease burden in *GRN* carriers, this SNP was denominated as the “protective SNP”. The major allele codes for threonine in amino acid position 185 in TMEM106B, whereas the minor allele codes for serine.

TMEM106B is a lysosomal type II transmembrane protein of unknown molecular function [23]. The overexpression of TMEM106B leads to distinctively enlarged endo-/lysosomal organelles in cultured cells with reduced proteolytic capabilities and changes in the lysosomal pH [7, 13]. The knockout of *Tmem106b* in mice leads to the formation of drastically enlarged axonal structures with LAMP1-positive vacuoles at the distal end of the axon initial segment of motorneurons and thalamic neurons [28]. The precise cause for these remarkable pathological abnormalities remains to be clarified, but several lines of evidence hint toward an impaired retrograde axonal transport of endo-/lysosomes, normally mediated by TMEM106B. Aged *Tmem106b*^*−/−*^ knockout mice develop generalized micro- and astrogliosis and Purkinje cell death, accompanied by signs of ataxia [40]. If challenged in experimental models for de- and remyelination, *Tmem106b*^*−/−*^ knockout mice show deficits in the process of axonal remyelination, compatible with the finding of a dominant *TMEM106B* mutation as the underlying cause for a rare form of hypomyelinating leukodystrophy [10, 38, 47]. Notably, the additional knockout of *Tmem106b*^*−/−*^ in *Grn*^*−/−*^ knockout mice has a tremendously deteriorating effect with severe motorneuron loss and strongly reduced life span, supporting a genetic interaction between the FTLD risk factors *TMEM106B* and *GRN* [11, 45, 46].

About one-third of the 274 amino acid long TMEM106B protein composes the cytosolic tail, followed by a single transmembrane domain and the remaining C-terminal highly N-glycosylated luminal domain [23]. Human and mouse TMEM106B are highly conserved with 96% amino acid identity. The murine protein is one single amino acid longer (275 amino acids) compared to the human protein. Artificial intelligence-based structure prediction predicts a β-sheet-rich globular fold of the C-terminal luminal domain, and a function as a lipid-binding protein was speculated due to the structural homology with an archaeal lipid-binding protein [25]. However, experimental evidence for such a function is lacking so far. Threonine/Serine 185 is located in the luminal domain in one of the predicted β-sheets. Very little is known about the possible effects of the amino acid exchange on the proteins’ function or biochemical properties: In cell-based overexpression studies, reduced stability of the S185 variant was observed [29], but the mechanisms leading to this reduced stability and finally lower TMEM106B levels under steady-state conditions remained unknown. Threonine/Serine 185 is located in close proximity to an occupied N-glycosylation site (N183), and it was speculated that the threonine/serine might have a subtle influence on the N-glycosylation pattern and usage of this particular site; however, no differences in the molecular weight of the two variants were apparent. Notably, upon overexpression of the threonine- or serine variant, no differences were observed in the lysosomal localization of TMEM106B [29].

Here we aimed to investigate the relevance and function of the T186S variant in vivo in a *Tmem106b* knockin mouse that harbors the serine in amino acid position 186 instead of the threonine in wildtype mice. We crossed these mice in the *Grn*^*−/−*^ knockout background to investigate the effect of the “protective variant” in a model that resembles some pathological features of human FTLD.

## Material & methods

### *Generation and genotyping of Tmem106b*^*T186S*^ knockin mice

For the generation of mice expressing *Tmem106b* with the p.T186S knockin, a single-guide RNA (sgRNA) was chosen after submitting the targeting region around exon 6 to the CRISPOR design tool (http://crispor.tefor.net; [18]). The template for transcription with the targeting sequence (TAAACGAGCCTTTCCAATCA) was generated by fill-in reaction with Klenow DNA Polymerase (Thermo Fisher Scientific). Transcription was performed using the HiScribe™ T7 High Yield RNA Synthesis Kit (#E2040S, New England Biolabs), with subsequent purification of the transcript with the MEGAClear™ Transcription Clean-Up Kit (#AM1908, Thermo Fisher Scientific), both according to the manufacturer’s instructions. A 120 bp repair template (Sigma-Aldrich) designed to knockin the p.T186S substitution and additional silent mutations to suppress further Cas9 cleavage activity had the following sequence: 5’-CCCTCAACTCCTGAATAGGCTTACCTGCTTCATATCAAGTGGGCCAATATTGCTTATGTTGTTTAAACGAGCTTTTCCAATCACTGTTTTTGAAAACTGGACTTGAGCAGTGATGTTTTC-3’. This donor DNA (1 µg/µL), sgRNA (600 ng/µL), and Cas9 protein (Alt-R® S.p. Cas9 Nuclease V3, #1,081,058, Integrated DNA Technologies (IDT), Leuven, Belgium) (500 ng/µL) in Gibco™ Opti-MEM™ (Thermo Fisher Scientific) were used for electroporation into one-cell-stage embryos derived from superovulated C57BL/6JUke mice using the NEPA 21 electroporator (Nepa Gene, Ichikawa-City, Japan; for settings, see [32]) and implanted into foster mice.

The *Tmem106b*^*T186S*^ knockin mice were genotyped by PCR using genomic tail DNA. Primers were designed to amplify a 478 bp fragment: Tmem106b_T186S_for: AAATGAGATATAGTTCCAAGTAAAGTCC and Tmem106b_T186S_rev: AGGATGAGGGATTTTCAGA. PCR products were enzymatically cleaned up using ExoSAP-IT™ (Applied Biosystems) for 15 min at 37 °C, following 15 min at 85 °C. Afterward, the purified samples were sent to Sanger sequencing (Eurofins Genomics).

### Animals/Animal husbandry

*Tmem106b* knockout mice generated by CRISPR/Cas9 and *Grn*^*−/−*^ knockout mice generated by targeted gene disruption were described before [21, 28]. Mice were housed under standard laboratory conditions with a 12 h light/dark cycle and constant room temperature and humidity. Food and water were available ad libitum. Experimental protocols including transcardial perfusion, were approved by the local German authorities (Ministerium für Energiewende, Landwirtschaft, Umwelt und ländliche Räume, Kiel, V 242–28,406/2020 (43–6/20)). Mixed cohorts of female and male animals were used throughout the study.

### Chemicals and antibodies

Analytical grade chemicals were purchased, if not stated otherwise, from Sigma-Aldrich (MO., USA). The following antibodies were used for immunofluorescence on brain sections: CD68 (1:500; rat monoclonal; clone FA-11 (MCA1957, AbD Serotec)), LAMP1 (1:500; rat monoclonal; clone 1D4B, Developmental Studies Hybridoma Bank (University of Iowa, Iowa City, IA, USA)), and Iba1 (1:500, rabbit polyclonal; GTX100042, Genetex). Fluorophore-conjugated secondary antibodies against the corresponding primary antibody species (AlexaFluor 488, AlexaFluor 594, and AlexaFluor 647) were purchased from Invitrogen/Molecular Probes and were diluted 1:500. HRP-coupled secondary antibodies were purchased from Dianova. The following primary antibodies were used for immunoblotting: TMEM106B (1:1000, rabbit monoclonal;E7H7Z, Cell Signaling Technology), and Na^+^/K^+^-ATPase (1:250, mouse monoclonal; clone a5, Developmental Studies Hybridoma Bank (University of Iowa, Iowa City, IA, USA).

### Preparation of brain lysates and immunoblotting

Brain lysates were prepared by homogenization of fresh or frozen brain material with 10–20 strokes at 1,000 rounds per minute using a Glass homogenizer (B.Braun type 853,202) in 15 volumes of lysis buffer (50 mM Tris–HCl, 50 mM NaCl, 1 × Complete™ protease inhibitor cocktail and 0,5% (w/v) Triton X-100). After homogenization, the lysates were ultrasonicated twice for 20 s at 4 °C using a Branson Sonifier 450 (level seven in a cup horn, Emerson Industrial Automation) and lysed on ice for approximately 30 min. The lysates were cleared at 16,000 × g for 15 min at 4 °C, and the protein concentration of the supernatant was determined using the Pierce BCA (bicinchoninic acid) Protein Assay Kit (Thermo Fisher Scientific) according to the manufacturer’s instructions. Protein lysates were prepared for SDS-PAGE in Laemmli sample buffer (125 mM Tris/HCl pH 6.8, 10% (v/v) glycerol, 1% (w/v) SDS, 1% (v/v) ß-mercaptoethanol and traces of bromophenol blue) and were denatured for 5 min at 95 °C. Western blot was carried out according to standard procedures. After washing the membranes in TBS-T buffer, horseradish peroxidase activity was detected by using an ImageQuant LAS 4000 (GE Healthcare). The intensity of the signal was quantified using Image J software. Before incubation with additional antibodies, the membranes were stripped using a glycine stripping buffer (100 mM glycine, 20 mM magnesium acetate, 50 mM potassium chloride). Incubations of 2 × 10 min at room temperature and gentle shaking were performed with glycine stripping buffer, followed by 2 × 10 min with PBS, and finally with 2 × 10 min TBS-T. Next, the membranes were incubated in 5% (w/v) milk powder in 1 × TBS-T buffer for 1 h at room temperature, followed by incubation with the first antibody.

### Nanostring analysis

Total RNA was isolated from frozen brains of 6-month-old mice using the Nucleospin® RNA Midi Kit from Machery & Nagel according to the manufacturer’s protocol. 50 ng of RNA was used for gene expression profiling using the nCounter Analysis from NanoString Technologies, Inc. (Glial Profiling Panel). The NanoString data were analyzed and normalized using nSolver™ software (version 4.0, NanoString Technologies, Inc.). RNA nCounts were normalized using the geometric mean of 8 housekeeping genes (*Aars, Ccdc127, Csnk2a2, Fam104a, Gusb, MtoI, Tada2b, and Xpnpep1*) using nSolver™ software.

For the pathway analysis, all differentially expressed genes with a *p*-value < 0.01 between wildtype and *Grn*^*−/−*^ or wildtype and *Grn*^*−/−*^ × *Tmem106b*^*T186S/T186S*^ were selected and used as input lists for the pathway enrichment analysis with the “Enrichr” online-tool [22].

### Immunofluorescence of brain sections

The mice were deeply anesthetized, followed by transcardial perfusion with 0.1 M phosphate buffer (PB) pH 7.4 and 4% paraformaldehyde (PFA) in PB. The brains were removed and post-fixed by immersion for another 4 h in 4% PFA. The PFA was removed and replaced by 30% sucrose (w/v) in 0.1 M PB. After the incubation of the brains overnight, 35 µm thick free-floating sagittal sections were cut with a Leica 9000 s sliding microtome (Leica, Wetzlar, Germany). The sections were blocked in blocking solutions (0.5% Triton-X 100, 4% normal goat serum in 0.1 M PB pH 7.4) and incubated in a blocking solution containing the primary antibody/antibodies at 4 °C overnight. After washing three times with wash solution (0.1 M PB pH 7.4 0.25% Triton X-100), sections were incubated for 90 min in secondary antibody in solution, washed two times again in wash solution containing 0,25% Triton X-100, and one time in wash solution without Triton X-100. Finally, the brain sections were mounted on glass slides and embedded in Mowiol/DABCO containing 1 mg/ml 4′,6-Diamidin-2-phenylindol (DAPI). The sections were analyzed with a Zeiss LSM 980 fluorescence microscope equipped with an automated stage and the ZEN 3.3 software. The fluorescence area of CD68, LAMP1 and autofluorescence was quantified using ImageJ software.

For three-dimensional reconstructions of microglia cells, 50 images were acquired in the Z-direction and assembled with the arivis Vision4D 3.3 software (Zeiss). The machine-learning module in the arivis Vision4D software was used to quantify the volume of the CD68-positive phagosomal compartment in individual microglia cells.

### Lysosomal enzyme activities

Aliquots of powdered mouse brain tissues were used for cathepsin D and L fluorescence-based activity assays (Abnova). The samples were homogenized in the appropriate lysis buffer provided by the manufacturer and incubated for 20 min on ice, followed by a 20 min centrifugation at 15,000 × g, 4 °C. The protein concentration was determined by BCA protein assay (Thermo Scientific Scientific), and equal amounts of protein were used for the activity assays. The assays were performed in black 96-well plates (FluoroNunc) at 37 °C for 30 min, according to the manufacturer’s protocol. Cleavage of the quenched fluorescence substrate was continuously measured as an increase of fluorescence signal by Fluoroskan Ascent FL plate reader (Labsystems). The relative enzyme activity was calculated for a period of time with linear substrate turnover.

### Lipid extraction

For lipid analysis, the cerebral cortex was dissected, weighed, and flash-frozen. 20 µl methanol per mg tissue and an internal standard were added to each sample and homogenized with 1.4 mm ceramic beads (shaken at 2.6 m/s for 30 s) using a Fisherbrand bead mill 24 homogenizer (Thermo Fisher Scientific). After centrifugation (20 min at 4 °C, 14,000 × g), the supernatant was transferred to a fresh tube, incubated for at least 1 h at − 20 °C, followed by an additional 20 min centrifugation (4,000 × g, 4 °C).

### Quantification of BMP using Q-TOF mass spectrometry

After lipid extraction and centrifugation, the supernatant was dried with nitrogen flow. The extracted lipids were dissolved in 100 µl of 100% ethanol. BMP was analyzed as previously described [35] with some modifications. BMP was separated on a Macherey & Nagel Nucleoshell HILIC column (50 × 4.6, 2.7 µm particle size) using an Agilent 1200 Series HPLC with a binary pump at a flow rate of 0.8 ml/min. The analysis was carried out with an Agilent 6530 Q-TOF LC/MS device. BMPs were measured in the positive ion mode; in this mode, BMPs form protonated adducts ([M + H] +). MS/MS spectra were recorded for the m/z values of the main molecular species of BMP. A product ion scan for one or two characteristic monoacylglycerol fragments for each molecular species was performed using the MassHunter Qualitative Analysis software.

## Results

### *The TMEM106B S186 variant shows a similar expression level as the T186 variant and is functional *in vivo

**Fig. 1:**
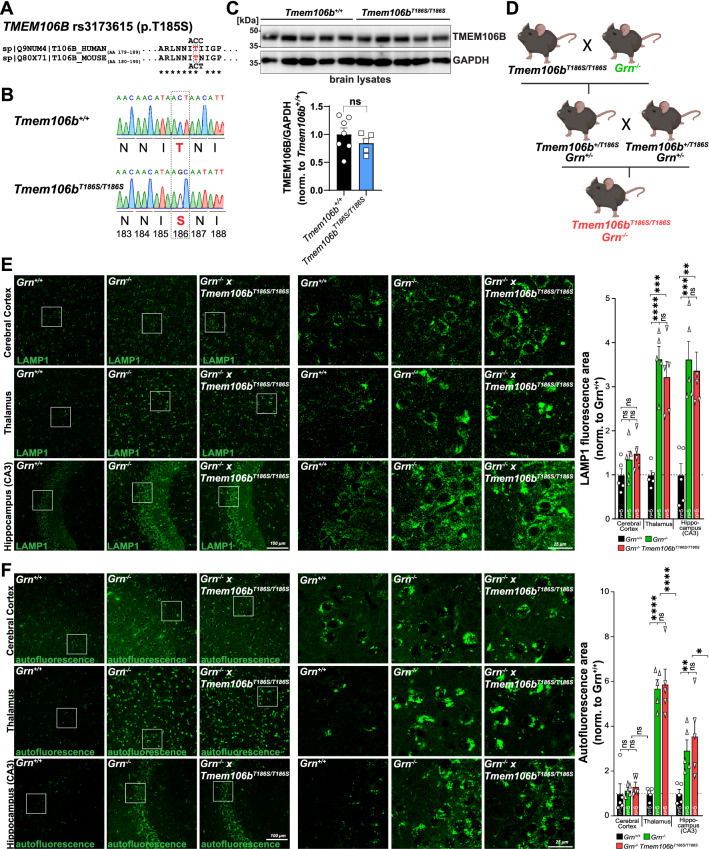
**A** Alignment of the protein sequence of human and mouse TMEM106B around the critical threonine (red) that is coded by the rs3173615 SNP in humans. T185 in humans corresponds to T186 in mice. The codon coding for the threonine is indicated. **B** Sequencing chromatogram of genomic tail DNA of a wildtype and a homozygous *Tmem106b*^*T186S/T186S*^ mouse with the codons for threonine 186 in the wildtype mouse and serin 186 (red) in the knockin mouse after gene editing of the “ACT” codon to “AGC”. A third base in the following codon is edited in the wobble-position without affecting the coded amino acid asparagine. **C** Immunoblot of brain lysates from wildtype and *Tmem106b*^*T186S/T186S*^ mice (n = 5–7, nine months old) with antibodies against TMEM106B and GAPDH as a loading control. The quantification of the TMEM106B signal is depicted (average of wildtype set as “1”). **D** Breeding scheme depicting the crossing of *Tmem106b*^*T186S/T186S*^ and *Grn*^*−/−*^ mice to obtain *Grn*^−/−^ × *Tmem106*^*T186S/T186S*^. **E** Representative images of immunofluorescence stainings of the cerebral cortex, thalamus, and the hippocampus (CA3 subfield) of a wildtype, *Grn*^*−/−*^, and *Grn*^*−/−*^ × *Tmem106b*^*T186S/T186S*^ with an antibody against LAMP1 (green). The quantification of the LAMP1-positive area/section is provided (n = 5, age: 6 months). **F** Representative images of autofluorescence recorded with laser excitation at 594 nm of the cerebral cortex, thalamus, and the hippocampus (CA3 subfield) of a wildtype, *Grn*^*−/−*^, and *Grn*^*−/−*^* × Tmem106b*^*T186S/T186S*^. The quantification of the autofluorescence-positive area/section is provided (n = 5. Age: 6 months)

Aiming to mimic the coding rs3173615 SNP in the human *TMEM106B* gene in mice, we compared the protein sequence of TMEM106B in both species. The critical serine 185 (in humans) is conserved between humans and mice and corresponds to serine 186 in mice, making it suitable to mimic the coding SNP (Fig. 1A). We used CRISPR/Cas9 technology to generate a knockin mouse line that expresses TMEM106B p.T186S corresponding to human TMEM106B p.T185S sgRNAs targeting murine *Tmem106b* exon six and a single-stranded DNA donor containing substitutions c.[557C > G;558 T > C], as well as silent substitutions to suppress targeting of the recombined template, were injected into one-cell stage mouse embryos and implanted into foster mice. The offspring of one mutation-positive founder mice were bred to homozygosity (*Tmem106b*^*T186S/T186S*^), and sequencing of genomic tail DNA confirmed the germline editing at the *Tmem106b* locus (Fig. 1B). Analysis of brain homogenates by immunoblot of 9-month-old wildtype and *Tmem106b*^*T186S/T186S*^ mice revealed no significant differences in the levels of TMEM106B (Fig. 1C). We did not observe any differences in the apparent molecular weight between the genotypes, which might suggest any differences in the N-glycosylation of TMEM106B.

To test if the T186S variant leads to altered function of TMEM106B in vivo, we used immunofluorescence staining of brain sections from 2-month-old mice and LAMP1 staining: *Tmem106b*^*−/−*^ knockout mice show prominent LAMP1-positive vacuoles in the facial motor nucleus as described before [28] (Additional File 1: Fig. S1A); such vacuoles are entirely absent in wildtype and *Grn*^*−/−*^ mice and importantly, no such vacuoles were observed in the facial motor nucleus of homozygous *Tmem106b*^*T186S/T186S*^ mice. Moreover, the crossing of *Tmem106b*^*T186S/T186S*^ mice with *Tmem106b*^*−/−*^ knockout mice (yielding compound heterozygote *Tmem106b*^*−/T186S*^ mice) (Additional File 1: Fig. S1B) followed by immunofluorescence analysis revealed a full rescue of the vacuolization phenotype seen in the homozygous knockout mice, indicating functionality of the TMEM106B S186 variant. In summary, our experiments did not reveal any difference in the steady state levels or function of the TMEM106B S186 variant.

### *The TMEM106B S186 variant does not ameliorate microgliosis, lipofuscin deposition, or lysosomal expansion in Grn*^*−/−*^* mice*

**Fig. 2:**
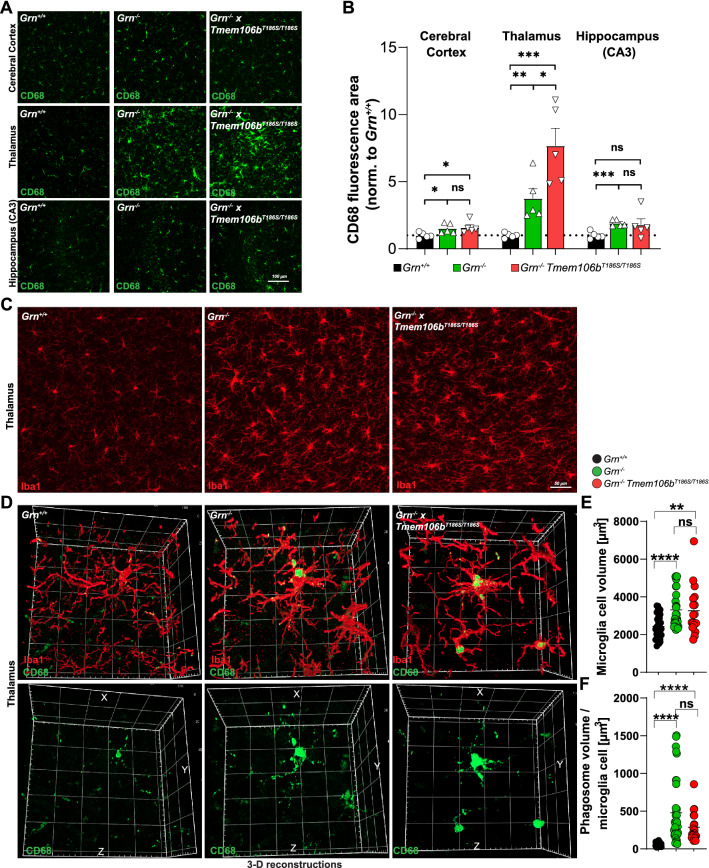
**A** Representative images of immunofluorescence stainings of the cerebral cortex, thalamus, and the hippocampus (CA3 subfield) of a wildtype, *Grn*^*−/−*^, and *Grn*^*−/−*^ × *Tmem106b*^*T186S/T186S*^ with an antibody against CD68 (green). **B** The quantification of the CD68-positive area/section is indicated (n = 5; Age: 6 months). **C** Representative images of immunofluorescence stainings of the thalamus of a wildtype, *Grn*^*−/−*^, and *Grn*^*−/−*^ × *Tmem106b*^*T186S/T186S*^ with an antibody against Iba1 (red). Age: 6 month. **D** Three-dimensional reconstructions from representative microglia cells of a wildtype, *Grn*^*−/−*^, and *Grn*^*−/−*^ × ^*T186S/T186S*^ mouse stained for Iba1 (red) and CD68 (green) from 50 images in the Z-direction of the thalamus (age: 6 months). **E** Volumetric quantification of the microglia cell volume of individual cells depicted from 3D reconstructed images. **F** Volumetric quantification of the CD68-positive phagosome compartment in individual Iba1-positive microglia cells depicted from 3D reconstructed images. **B, E, F** *p < 0.05; **p < 0.01; ****p < 0.001; *ns* Not significant

The rs3173615 SNP in *TMEM106B* confers its highest protective effect in *GRN* carriers and protects them from diseases [3]. To test if the coding variant is protective in an FTLD mouse model, we crossed the *Tmem106b*^*T186S/T186S*^ mice with *Grn*^*−/−*^ knockout mice to obtain *Grn*^*−/−*^ knockout mice in the rs3173615 SNP background *Tmem106b* S186 and compared them with *Grn*^*−/−*^ knockout mice in the *Tmem106b* wildtype background (Fig. 1D). As a readout for a possible protective effect, we used immunofluorescence with established markers (LAMP1, autofluorescence, CD68/Iba1) that clearly distinguish *Grn*^*−/−*^ knockout mice from wildtype mice and chose an age of 6 months with robust differences between wildtype and *Grn*^*−/−*^ knockout mice [1, 17, 41]. First, we analyzed LAMP1 as a marker for lysosomes [41]. We observed a clear increase in the LAMP1 signal intensity in the thalamus and the hippocampus of *Grn*^*−/−*^ mice compared to wildtype mice but no major differences between *Grn*^*−/−*^ mice (in the wildtype *Tmem106b* background) and *Grn*^*−/−*^ × *Tmem106b*^*T186S/T186S*^ mice (Fig. 2D). In the cerebral cortex, we observed a similar trend which, however, did not reach statistical significance. Next, we compared the levels of autofluorescent lipofuscin by histology, known to accumulate in the brains of *Grn*^*−/−*^ mice in an age-dependent manner [1]. Similar to LAMP1, lipofuscin was clearly increased in the thalamus and the hippocampus of *Grn*^*−/−*^ mice compared to wildtype animals, but comparable between *Grn*^*−/−*^ mice and *Grn*^*−/−*^ × *Tmem106b*^*T186S/T186S*^ mice (Fig. 2E). In summary, these data indicate that the T186S TMEM106B variant cannot ameliorate the levels of LAMP1 or accumulation of lipofuscin in the *Grn*^*−/−*^ mouse model.

### *The TMEM106B S186 variant does not ameliorate microgliosis and neuroinflammation-related transcriptomic changes in Grn*^*−/−*^* mice*

Next, we focussed our analyses on microgliosis, which is a prominent and early pathological finding of *Grn*^*−/−*^ mice [1, 17, 41]. Immunofluorescence staining of brain sections from 6-month-old wildtype, *Grn*^*−/−*^ and *Grn*^*−/−*^ × *Tmem106b*^*T186S/T186S*^ mice with an antibody against the phagosomal marker CD68 revealed a clear increase of the CD68 signal in different brain regions (cerebral cortex, thalamus, hippocampus) in *Grn*^*−/−*^ mice (as described previously, [41]), and, likewise, similarly in *Grn*^*−/−*^ × *Tmem106b*^*T186S/T186S*^ mice (Fig. 2A). Image quantification confirmed these observed differences and revealed a statistically significant increase in the CD68 area on the sections of the hippocampus and the cerebral cortex between *Grn*^*−/−*^ mice compared to wildtype mice but not compared to *Grn*^*−/−*^ × *Tmem106b*^*T186S/T186S*^. In the thalamus, *Grn*^*−/−*^ × *Tmem106b*^*T186S/T186S*^ showed even stronger CD68 immunoreactivity compared to *Grn*^*−/−*^ mice (Fig. 2B). Immunofluorescence staining of brains from 6-month-old animals with Iba1, a microglia-specific marker that is localized at the plasma membrane of the microglia cells and allows the assessment of the cell morphology, revealed the hypertrophy of microglia cells in the thalamus of *Grn*^*−/−*^ and *Grn*^*−/−*^ × *Tmem106b*^*T186S/T186S*^ mice. Microglia in the thalamus of *Grn*^*−/−*^ and *Grn*^*−/−*^ × *Tmem106b*^*T186S/T186S*^ mice were enlarged with a thicker cell body and thickened branches (Fig. 2C). This microglia hypertrophy became even more evident when we analyzed images from three-dimensional reconstructions of Iba1- and CD68-stained microglia cells (Fig. 2D, Additional File 1: Fig. S2). The quantification of the cell volume of individual microglia cells revealed a robust increase in both *Grn*^*−/−*^* and Grn*^*−/−*^ × *Tmem106b*^*T186S/T186S*^ mice compared to wildtype mice (Fig. 2E). The quantification of the CD68-positive phagosomal compartment in these individual microglia cells showed, in agreement with the increased cell volume, a pronounced enlargement of phagosomes in both *Grn*^*−/−*^ and *Grn*^*−/−*^ × *Tmem106b*^*T186S/T186S*^ mice compared to wildtype mice (Fig. 2F).

**Fig. 3:**
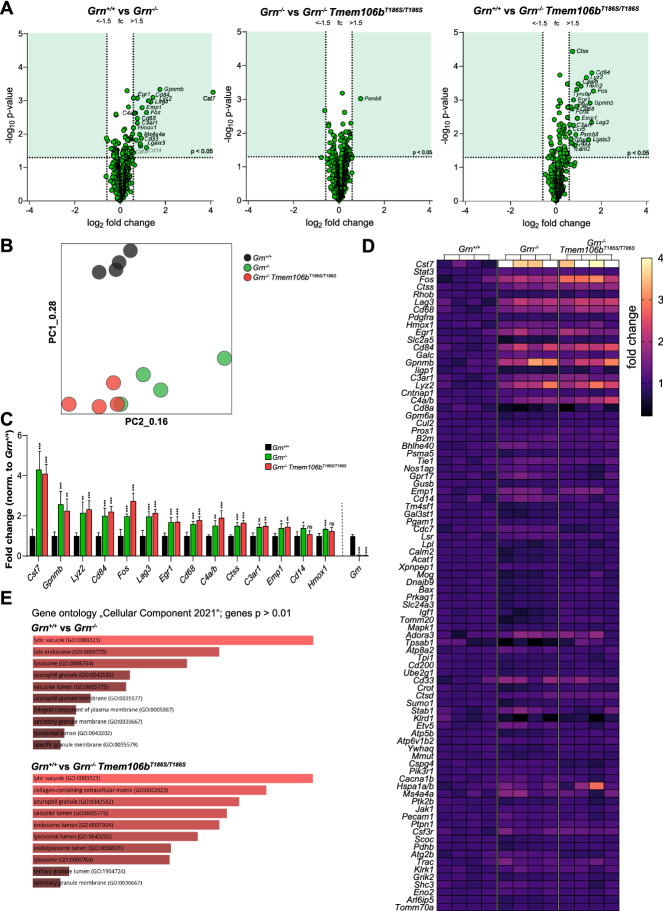
**A** Volcano plot presentation of the differently expressed transcripts between wildtype vs. *Grn*^*−/−*^ (left panel), *Grn*^*−/−*^ vs. *Grn*^*−/−*^ × *Tmem106b*^*T186S/T186S*^ and wildtype vs *Grn*^*−/−*^ × *Tmem106b*^*T186S/T186S*^ mice (n = 4, each genotype) in RNA isolated from 6-month-old mice. The thresholds for transcripts reaching statistically significant differences (fold change (fc) < − 1.5 / > 1.5; *p* < 0.05) are indicated (dotted lines), and the quadrants with transcripts reaching the threshold are colored in green. *Grn* as the top-down-regulated transcript in *Grn*^*−/−*^ and *Grn*^*−/−*^ × *Tmem106b*^*T186S/T186S*^ mice was excluded in the volcano-plot representation. **B** Principal component analysis (PCA) of the 12 total brain samples analyzed by Nanostring nCounter “Glia Profiling” panel. Every individual point represents one individual biological replicate: Wildtype = black, *Grn*^*−/−*^ = green *Grn*^*−/−*^ × *Tmem106b*^*T186S/T186S*^ = red. Note that the wildtype group can be clearly separated from the other two groups, while there is an overlap between *Grn*^*−/−*^ and *Grn*^*−/−*^ × *Tmem106b*^*T186S/T186S*^. **C** Fold changes of the top 14 transcripts differentially expressed between wildtype and *Grn*^*−/−*^ mice determined by NanoString nCounter analysis. Statistics indicate significant differences between wildtype and *Grn*^*−/−*^ / wildtype and *Grn*^*−/−*^ × *Tmem106b*^*T186S/T186S*^ mice. For non of the selected genes, any significant differences were found between *Grn*^*−/−*^ and wildtype and *Grn*^*−/−*^ × *Tmem106b*^*T186S/T186S*^. *p < 0.05; **p < 0.01; ***p < 0.001; ns = not significant; (n = 4, each genotype). **D** Heatmap of 86 gene transcripts analyzed by NanoString nCounter analysis in wildtype, *Grn*^*−/−*^, *Grn*^*−/−*^ × *Tmem106b*^*T186S/T186S*^ mice (n = 4, each genotype) from total brain RNA isolated from 6-month-old mice. The expression-corrected and housekeeping gene-normalized RNA counts for each gene and sample were normalized to the mean value of the wildtype animals. *Grn* was excluded from this representation. **E** Pathway enrichment of differentially expressed genes (*p* < 0.01) between wildtype and *Grn*^*−/−*^ mice (upper panel) and wildtype and *Grn*^*−/−*^ × *Tmem106b*^*T186S/T186S*^ mice (lower panel) by the “Enrichr” tool. The top-enriched terms of the gene ontology term “Cellular Component 2021” are depicted

We next analyzed possible transcriptomic differences between wildtype, *Grn*^*−/−*^ mice, and *Grn*^*−/−*^ × *Tmem106b*^*T186S/T186S*^ mice at the age of 6 months by the Nanostring nCounter technology and the predesigned “Glial Profiling” panel. The nCounter technology allows sensitive gene expression analysis without the need for reverse transcription or RNA amplification. We used, instead of applying global or bulk sequencing, the “Glial profiling”-panel covering a total of 770 genes related to neuroinflammation, cell stress/damage response, and glia homeostasis for the transcriptomic analysis (Additional File 2: Table S1). The comparison between wildtype and *Grn*^*−/−*^ mice revealed the expected and previously described transcriptomic differences [15, 16, 31]: top differentially expressed genes were characteristic for disease-associated microglia with *Cst7, Gpnmb, Lyz2,* and *Cd68* among the genes with the highest upregulation in total brain RNA derived from *Grn*^*−/−*^ mice (Fig. 3A). The comparison of the main effects on the transcriptome changes by principal component analysis of four animals per genotype revealed a clear separation of the four wildtype mice from the *Grn*^*−/−*^ mice and *Grn*^*−/−*^ × *Tmem106b*^*T186S/T186S*^ mice (Fig. 3B). The latter two groups were separated, however; they still showed overlap, suggesting a more similar transcriptome. In contrast, no major gene expression changes could be observed between *Grn*^*−/−*^ mice and *Grn*^*−/−*^ × *Tmem106b*^*T186S/T186S*^ mice (Fig. 3B). This lack of an effect (of the *Tmem106b*^*T186S/T186S*^ alleles) was apparent for any of the individual genes with the greatest fold changes between wildtype and *Grn*^*−/−*^ mice and was observed for the entire set of analyzed transcripts (Fig. 3C and D). Pathway enrichment analysis of differentially expressed genes showed a clear enrichment of “Gene ontology Molecular Function” terms related to lysosomes, e.g., “lytic vacuole”, “vacuolar lumen”, and “lysosome” when wildtype and *Grn*^*−/−*^ mice were compared or when wildtype and *Grn*^*−/−*^ × *Tmem106b*^*T186S/T186S*^ were compared, suggesting a very similar overall transcriptomic response (Fig. 3E).

**Fig. 4:**
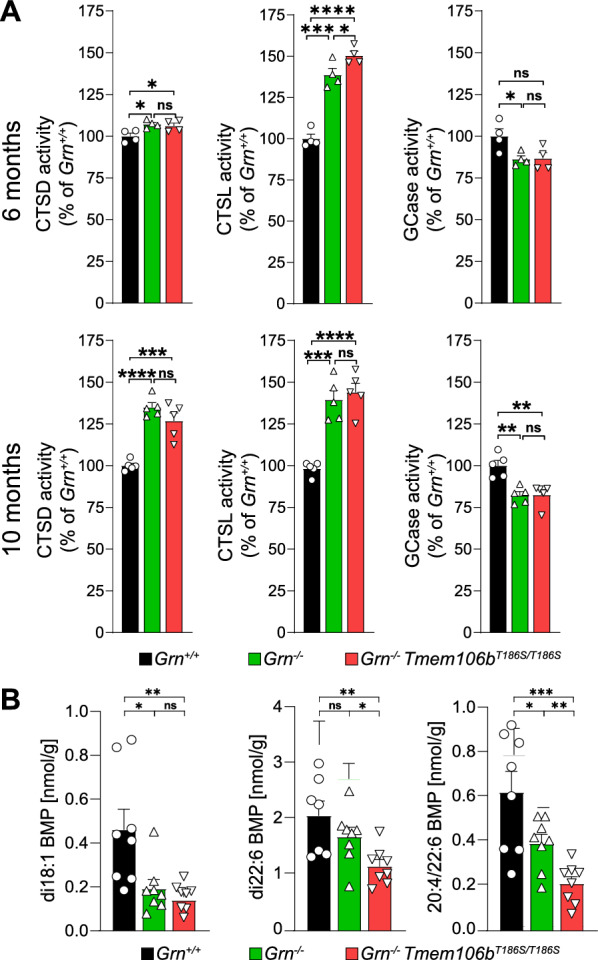
**A** Specific enzymatic activity of the three lysosomal enzymes cathepsin D (CTSD), cathepsin L (CTSL), and β-Glucocerebrosidase (GCase) in brain lysates of 6-month-old wildtype, *Grn*^*−/−*^, *Grn*^*−/−*^ × *Tmem106b*^*T186S/T186S*^ mice (n = 4, each genotype) (upper panel) and 10-month-old mice (lower panel). **B** The levels of the BMP species with different fatty acid compositions (di18:1, di22:6, and 20:4/22:6) were quantified in lipid extracts of the brain from 6-month-old wildtype, *Grn*^*−/−*^, *Grn*^*−/−*^ × *Tmem106b*^*T186S/T186S*^ mice (n = 8, each genotype) by mass spectrometry

The deficiency of progranulin affects the specific activity of several lysosomal enzymes, including cathepsin D (CTSD) and β-glucocerebrosidase (GCase) in the brain either by directly mediating their proteolytic processing or by affecting the general function of lysosomes [2, 4, 16, 27, 48, 49]. However, it also has been reported that lysosomal cathepsin expression and activity are elevated by progranulin deficiency [15, 16], presumably as an attempt to rescue lysosomal dysfunction. Possibly related to these changes in the specific activity of lysosomal enzymes, the lipidome in *Grn*^*−/−*^ mice is significantly changed with a clear decrease in the levels of the endo-/lysosome enriched lipid bis(monoacylglycerol)phosphate (BMP) [27]. To test if the S186 variant of TMEM106B affects these phenotypic differences between wildtype and *Grn*^*−/−*^ mice, we determined the specific activity of CTSD, CTSL, and GCase in total brain lysates of 6-month-old mice (Fig. 4A). While we observed statistically significant increases in the activities of CTSD and CTSL, the activity of GCase was decreased in *Grn*^−/−^ mice, as observed before [2, 4, 16, 27, 48, 49]. In contrast, no differences could be observed between *Grn*^*−/−*^ mice and *Grn*^*−/−*^ × *Tmem106b*^*T186S/T186S*^ mice in the enzymatic activity of CTSD or GCase, and only a modest but statistically significant increase in CTSL activity (Fig. 4A). Very similar but even more pronounced effects were observed in brain lysates of mice at the age of 10 months: CTSD and CTSL were significantly increased compared to wildtype animals with a higher difference compared to the 6-month-old mice, and no differences were observed between *Grn*^*−/−*^ mice and *Grn*^*−/−*^ × *Tmem106b*^*T186S/T186S*^ mice. GCase activity was, similar to 6 months old animals, decreased in brain lysate of *Grn*^*−/−*^ mice and *Grn*^*−/−*^ × *Tmem106b*^*T186S/T186S*^ mice compared to wildtype animals (Fig. 4A). No differences were observed between *Grn*^*−/−*^ mice and *Grn*^*−/−*^ × *Tmem106b*^*T186S/T186S*^ mice. Finally, we quantified the levels of three BMP lipid species with different fatty acid chain compositions (di18:1, di22:6, and 20:4/22:6), previously shown to be reduced in the brain of *Grn*^*−/−*^ mice [27] (Fig. 4B). A significant decrease of di18:1BMP and 20:4/22:6BMP was observed, while the trend was similar for di22:6BMP but did not reach statistical significance. Similarly, all three lipid species were statistically significantly reduced in *Grn*^*−/−*^ × *Tmem106b*^*T186S/T186S*^ mice compared to wildtype mice (Fig. 4B). In conclusion, the T186S amino acid change in TMEM106B does neither affect changes in lysosomal enzyme activity nor the changes of the BMP species in brain lysates.

## Discussion

FTLD, the second-most common early-onset dementia, is a highly heritable disorder, with around 40% of the patients having a familial history, indicating a high genetic contribution to the disease risk [34]. In an effort to identify genetic risk factors for FTLD, non-coding SNPs and a nonsynonymous coding SNP in *TMEM106B* have been associated with the risk of the major neuropathological subtype FTLD-TDP [43]. Interestingly, the association of the most significantly associated SNPs is greatest in people with *GRN*-related FTLD-TDP in *GRN* carriers versus in non-*GRN* carriers [43]. How TMEM106B affects the disease outcome in FTLD-TDP / FTLD-*GRN*, however, still remains enigmatic. Of particular interest in this regard is the question if the nonsynonymous coding SNP rs3173615 (coding for threonine in the major allele and serine in the minor, “protective” allele) directly affects TMEM106B’s function or properties. We addressed this question in vivo by generating a *Tmem106b* knockin mouse harboring the protective minor SNP-coded threonine, in order to dissect the complex problem how variants in *Tmem106b* affect the course of disease in *Grn*^*−/−*^ mice. With our knockin mouse we are able to test if the coding variant alone is sufficient to modulate the *GRN* phenotype, avoiding the complex interplay with other SNPs that might e.g. affect the expression levels. Overall, with all limitations of our study (see below), our data do not support a critical function of the amino acid exchange on TMEM106B’s biochemical properties or function. Homozygous *Tmem106b*^*T186S/186S*^ mice are indistinguishable from wildtype mice and lack any signs of the vacuolization phenotype observed in the *Tmem106b*^*−/−*^ knockout mice [28], indicating that the protein remains at least partially functional. Our analyses did not reveal any differences in the steady state levels of TMEM106B in total brain lysates, as could have been expected from previous, cell-based studies in which TMEM106B with the T or S variant was ectopically overexpressed under strong promoters [29]. In our in vivo setup, *Tmem106b* is expressed under the control of the endogenous promoter, preventing artificial pleiotropic effects on gene expression or abnormal endo-/lysosmal function. We did also not observe differences in the molecular weight of the TMEM106B S or T variants and, therefore, any differences in N-glycosylation, which might explain differences in stability. Whether the S186 variant is indeed less stable, therefore, is questionable.

When we crossed the *Tmem106b*^*T186S/186S*^ mice into the *Grn*^*−/−*^ background, we did not observe any protective effect. Even though our study might be underpowered and we used only relatively small cohorts, we did not observe an improved phenotype in any of the investigated parameters compared to *Grn*^*−/−*^ in the *Tmem106b* wildtype background. In contrast, for a few datapoints (CD68 signal in the thalamus, BMP levels) the effects were even more pronounced, even though it is not clear if this is caused by low number of analyzed animals; however, these data clearly show that *Tmem106B* T186S is not protective. A clear drawback of our study aiming to evaluate the functional consequences of the protective *TMEM106B* SNP in *GRN*-mediated FTLD is the model we used: In humans subjects, the great majority of patients bear heterozygous loss-of-function *GRN* mutations leading to haploinsufficiency of Progranulin [3]. Homozygous *GRN* mutations typically lead to the childhood to adolescent lysosomal storage disease “Neuronal ceroid lipofuscinosis type 11” (CLN11) [39]. Only recently, late-onset FTLD patients with homozygous *GRN* mutations were identified [19]. Thus, homozygous *Grn*^*−/−*^ knockout mice are not an appropriate model for human disease. Heterozygous *Grn*^±^ mice, on the other hand, show no robust phenotypic alterations compared to wildtype animals [21], excluding them from analyzing the effect of the TMEM106B “protective SNP” in the “haploinsufficiency” situation. The interaction between *TMEM106B* and *GRN* is still inadequately understood: while there is no evidence for direct physical interaction of the two proteins in lysosomes, human genetics with the clear association of *TMEM106B* variants/SNPs in FTLD-*GRN* patients and the mouse genetics (the *Tmem106b*^*−/−*^ knockout markedly exacerbates the *Grn*^*−/−*^ knockout phenotype) clearly suggest a genetic interaction between the two genes. However, we can not exclude that the TMEM106B SNPs only confer their protective function if one intact *GRN* allele still remains functional and progranulin is present in lysosomes. The clear difference between the characteristics of the pathology of the *Grn* mouse models and human FTLD is an intrinsic problem for studies like ours and is a natural limitation. The fact that the additional knockout of *Tmem106b* in the *Grn*^*−/−*^ mouse [45] has such a devastating effect indicates that the interaction between *TMEM106B* and *GRN* is conserved between humans and mice, makes it unlikely that the different species explain the lack of any effect observed in our study, though we cannot fully rule out that the human TMEM106B version carrying the protective variant (full human *TMEM106B* knockin model) may potentially lead to different outcomes if expressed in *Grn*^*−/−*^ mice. However, mice have a much shorter life expectancy, and possibly TMEM106B (and the two different coding variants with threonine or serine) modulates mostly aging-related effects far beyond the life expectancy of a laboratory mouse. In this regard, it should be noted that TMEM106B regulates differential aging [33]. Alternative, complementary approaches like modeling FTLD in *GRN*-carrier derived congenic induced pluripotent stem cell (iPSC)-derived neurons or microglia, and CRISPR-mediated manipulation of the *TMEM106B* locus might be helpful to determine if the “protective SNP” has any beneficial effect in the presence of an intact *GRN* allele in a human cellular system.

The critical question which we could not conclusively address is how variants in *TMEM106B* modulate disease risk in *GRN*-carriers. While the rs3173615 SNP is the only coding variant, it was self-evident, assuming that it affects the protein’s function or biochemical properties directly. (Alternative: The SNP rs3173615 is the only coding variant, so it was reasonable to assume that it directly affects the function or biochemical properties of the protein. However, alternative scenarios could explain how variants in *TMEM106B* affect disease risk: It was previously shown that one of the non-coding *TMEM106B* SNPs increases the recruitment of the chromatin-organizing protein CCCTC-binding factor (CTCF) downstream of *TMEM106B*, thereby affecting its gene expression, transcript and protein levels [13]. Indeed, differential TMEM106B transcript levels depending on the rs1990622 SNP (which is in high linkage disequilibrium with rs3173615) were observed already in the initial study that identified *TMEM106B* as an FTLD risk gene [39], and the levels of TMEM106B directly affect lysosomal size, morphology, and positioning [9, 13, 25, 37]. Our experiments support such disease-modulating effects, e.g., on the transcription of *TMEM106B*, unrelated to the amino acid exchange, as the likely explanation for the modifying effect on disease onset and risk.

## Supplementary Information

**Additional file 1: Fig. S1**. **A** Representative images of immunofluorescence stainings of the facial motor nucleus of a wildtype, *Tmem106b*^−/−^, *Tmem106b*^*T186S/T186S*^ mouse with an antibody against LAMP1(upper panel green, lower panel white). Nuclei are stained with DAPI (upper panel, blue). **B** Breeding scheme depicting the crossing of *Tmem106b*^*+/T186S*^ and *Tmem106b*^−/−^ to produce *Tmem106b*^−/T186S^ with one knockout allele and one knockin allele. **C** Representative images of immunofluorescence stainings of the facial motor nucleus of a *Tmem106b*^*−/T186S*^ mouse with an antibody against LAMP1 (upper panel green, lower panel white). Nuclei are stained with DAPI (upper panel, blue).
**Additional file 1: Fig. S2**. Three-dimensional reconstructions of individual Iba1 stained microglia cells of the thalamus of* Grn*^+/+^, *Grn*^−/−^ and *Grn*^−/−^ *T﻿mem106b*^*T186S/T186S*^ mice (pseudocolored, three examples/genotype) used for cell-volume calculation. The panel on the right depicts overview images used for quantification. 50 images were acquired in the Z-direction and assembled with the arivis Vision4D 3.3 software (Zeiss).

**Additional file 2: Table S1**. Full list of the gene expression data from the Nanostring nCounter analysis of brain RNA.

## Abbreviations

BMP: Bis(monoacylglycerol)phosphate
FTLD: Frontotemporal lobar degeneration
GWAS: Genome-wide association studies
SNP: Single nucleotide polymorphism

## References

[1] Ahmed Z, Sheng H, Xu YF, Lin WL, Innes AE, Gass J, Yu X, Wuertzer CA, Hou H, Chiba S (2010). Accelerated lipofuscinosis and ubiquitination in granulin knockout mice suggest a role for progranulin in successful aging. Am J Pathol.

[2] Arrant AE, Roth JR, Boyle NR, Kashyap SN, Hoffmann MQ, Murchison CF, Ramos EM, Nana AL, Spina S, Grinberg LT (2019). Impaired beta-glucocerebrosidase activity and processing in frontotemporal dementia due to progranulin mutations. Acta Neuropathol Commun.

[3] Baker M, Mackenzie IR, Pickering-Brown SM, Gass J, Rademakers R, Lindholm C, Snowden J, Adamson J, Sadovnick AD, Rollinson S (2006). Mutations in progranulin cause tau-negative frontotemporal dementia linked to chromosome 17. Nature.

[4] Beel S, Moisse M, Damme M, De Muynck L, Robberecht W, Van Den Bosch L, Saftig P, Van Damme P (2017). Progranulin functions as a cathepsin D chaperone to stimulate axonal outgrowth in vivo. Hum Mol Genet.

[5] Bellenguez C, Kucukali F, Jansen IE, Kleineidam L, Moreno-Grau S, Amin N, Naj AC, Campos-Martin R, Grenier-Boley B, Andrade V (2022). New insights into the genetic etiology of Alzheimer's disease and related dementias. Nat Genet.

[6] Bieniek KF, Ross OA, Cormier KA, Walton RL, Soto-Ortolaza A, Johnston AE, DeSaro P, Boylan KB, Graff-Radford NR, Wszolek ZK (2015). Chronic traumatic encephalopathy pathology in a neurodegenerative disorders brain bank. Acta Neuropathol.

[7] Busch JI, Unger TL, Jain N, Tyler Skrinak R, Charan RA, Chen-Plotkin AS (2016). Increased expression of the frontotemporal dementia risk factor TMEM106B causes C9orf72-dependent alterations in lysosomes. Hum Mol Genet.

[8] Chang A, Xiang X, Wang J, Lee C, Arakhamia T, Simjanoska M, Wang C, Carlomagno Y, Zhang G, Dhingra S (2022). Homotypic fibrillization of TMEM106B across diverse neurodegenerative diseases. Cell.

[9] Chen-Plotkin AS, Unger TL, Gallagher MD, Bill E, Kwong LK, Volpicelli-Daley L, Busch JI, Akle S, Grossman M, Van Deerlin V (2012). TMEM106B, the risk gene for frontotemporal dementia, is regulated by the microRNA-132/212 cluster and affects progranulin pathways. J Neurosci.

[10] Feng T, Luan L, Katz II, Ullah M, Van Deerlin VM, Trojanowski JQ, Lee EB, Hu F (2022). TMEM106B deficiency impairs cerebellar myelination and synaptic integrity with Purkinje cell loss. Acta Neuropathol Commun.

[11] Feng T, Mai S, Roscoe JM, Sheng RR, Ullah M, Zhang J, Katz II, Yu H, Xiong W, Hu F (2020). Loss of TMEM106B and PGRN leads to severe lysosomal abnormalities and neurodegeneration in mice. EMBO Rep.

[12] Finch N, Carrasquillo MM, Baker M, Rutherford NJ, Coppola G, Dejesus-Hernandez M, Crook R, Hunter T, Ghidoni R, Benussi L (2011). TMEM106B regulates progranulin levels and the penetrance of FTLD in GRN mutation carriers. Neurology.

[13] Gallagher MD, Posavi M, Huang P, Unger TL, Berlyand Y, Gruenewald AL, Chesi A, Manduchi E, Wells AD, Grant SFA (2017). A dementia-associated risk variant near TMEM106B alters chromatin architecture and gene expression. Am J Hum Genet.

[14] Gallagher MD, Suh E, Grossman M, Elman L, McCluskey L, Van Swieten JC, Al-Sarraj S, Neumann M, Gelpi E, Ghetti B (2014). TMEM106B is a genetic modifier of frontotemporal lobar degeneration with C9orf72 hexanucleotide repeat expansions. Acta Neuropathol.

[15] Gotzl JK, Brendel M, Werner G, Parhizkar S, Sebastian Monasor L, Kleinberger G, Colombo AV, Deussing M, Wagner M, Winkelmann J (2019). Opposite microglial activation stages upon loss of PGRN or TREM2 result in reduced cerebral glucose metabolism. EMBO Mol Med.

[16] Gotzl JK, Colombo AV, Fellerer K, Reifschneider A, Werner G, Tahirovic S, Haass C, Capell A (2018). Early lysosomal maturation deficits in microglia triggers enhanced lysosomal activity in other brain cells of progranulin knockout mice. Mol Neurodegener.

[17] Gotzl JK, Mori K, Damme M, Fellerer K, Tahirovic S, Kleinberger G, Janssens J, van der Zee J, Lang CM, Kremmer E (2014). Common pathobiochemical hallmarks of progranulin-associated frontotemporal lobar degeneration and neuronal ceroid lipofuscinosis. Acta Neuropathol.

[18] Haeussler M, Schonig K, Eckert H, Eschstruth A, Mianne J, Renaud JB, Schneider-Maunoury S, Shkumatava A, Teboul L, Kent J (2016). Evaluation of off-target and on-target scoring algorithms and integration into the guide RNA selection tool CRISPOR. Genome Biol.

[19] Huin V, Barbier M, Bottani A, Lobrinus JA, Clot F, Lamari F, Chat L, Rucheton B, Fluchere F, Auvin S (2020). Homozygous GRN mutations: new phenotypes and new insights into pathological and molecular mechanisms. Brain.

[20] Jiang YX, Cao Q, Sawaya MR, Abskharon R, Ge P, DeTure M, Dickson DW, Fu JY, Ogorzalek Loo RR, Loo JA (2022). Amyloid fibrils in FTLD-TDP are composed of TMEM106B and not TDP-43. Nature.

[21] Kayasuga Y, Chiba S, Suzuki M, Kikusui T, Matsuwaki T, Yamanouchi K, Kotaki H, Horai R, Iwakura Y, Nishihara M (2007). Alteration of behavioural phenotype in mice by targeted disruption of the progranulin gene. Behav Brain Res.

[22] Kuleshov MV, Jones MR, Rouillard AD, Fernandez NF, Duan Q, Wang Z, Koplev S, Jenkins SL, Jagodnik KM, Lachmann A (2016). Enrichr: a comprehensive gene set enrichment analysis web server 2016 update. Nucleic Acids Res.

[23] Lang CM, Fellerer K, Schwenk BM, Kuhn PH, Kremmer E, Edbauer D, Capell A, Haass C (2012). Membrane orientation and subcellular localization of transmembrane protein 106B (TMEM106B), a major risk factor for frontotemporal lobar degeneration. J Biol Chem.

[24] Lattante S, Le Ber I, Galimberti D, Serpente M, Rivaud-Pechoux S, Camuzat A, Clot F, Fenoglio C, Scarpini E, Brice A (2014). Defining the association of TMEM106B variants among frontotemporal lobar degeneration patients with GRN mutations and C9orf72 repeat expansions. Neurobiol Aging.

[25] Levine TP (2022). TMEM106B in humans and Vac7 and Tag1 in yeast are predicted to be lipid transfer proteins. Proteins.

[26] Li Z, Farias FHG, Dube U, Del-Aguila JL, Mihindukulasuriya KA, Fernandez MV, Ibanez L, Budde JP, Wang F, Lake AM (2020). The TMEM106B FTLD-protective variant, rs1990621, is also associated with increased neuronal proportion. Acta Neuropathol.

[27] Logan T, Simon MJ, Rana A, Cherf GM, Srivastava A, Davis SS, Low RLY, Chiu CL, Fang M, Huang F (2021). Rescue of a lysosomal storage disorder caused by Grn loss of function with a brain penetrant progranulin biologic. Cell.

[28] Luningschror P, Werner G, Stroobants S, Kakuta S, Dombert B, Sinske D, Wanner R, Lullmann-Rauch R, Wefers B, Wurst W (2020). The FTLD risk factor TMEM106B regulates the transport of lysosomes at the axon initial segment of motoneurons. Cell Rep.

[29] Nicholson AM, Finch NA, Wojtas A, Baker MC, Perkerson RB, Castanedes-Casey M, Rousseau L, Benussi L, Binetti G, Ghidoni R (2013). TMEM106B p. T185S regulates TMEM106B protein levels: implications for frontotemporal dementia. J Neurochem.

[30] Nicholson AM, Rademakers R (2016). What we know about TMEM106B in neurodegeneration. Acta Neuropathol.

[31] Reifschneider A, Robinson S, van Lengerich B, Gnorich J, Logan T, Heindl S, Vogt MA, Weidinger E, Riedl L, Wind K (2022). Loss of TREM2 rescues hyperactivation of microglia, but not lysosomal deficits and neurotoxicity in models of progranulin deficiency. Embo J.

[32] Remy S, Chenouard V, Tesson L, Usal C, Menoret S, Brusselle L, Heslan JM, Nguyen TH, Bellien J, Merot J (2017). Generation of gene-edited rats by delivery of CRISPR/Cas9 protein and donor DNA into intact zygotes using electroporation. Sci Rep.

[33] Rhinn H, Abeliovich A (2017). Differential aging analysis in human cerebral cortex identifies variants in TMEM106B and GRN that regulate aging phenotypes. Cell Syst.

[34] Rohrer JD, Guerreiro R, Vandrovcova J, Uphill J, Reiman D, Beck J, Isaacs AM, Authier A, Ferrari R, Fox NC (2009). The heritability and genetics of frontotemporal lobar degeneration. Neurology.

[35] Scherer M, Schmitz G, Liebisch G (2010). Simultaneous quantification of cardiolipin, bis(monoacylglycero)phosphate and their precursors by hydrophilic interaction LC-MS/MS including correction of isotopic overlap. Anal Chem.

[36] Schweighauser M, Arseni D, Bacioglu M, Huang M, Lovestam S, Shi Y, Yang Y, Zhang W, Kotecha A, Garringer HJ (2022). Age-dependent formation of TMEM106B amyloid filaments in human brains. Nature.

[37] Schwenk BM, Lang CM, Hogl S, Tahirovic S, Orozco D, Rentzsch K, Lichtenthaler SF, Hoogenraad CC, Capell A, Haass C (2014). The FTLD risk factor TMEM106B and MAP6 control dendritic trafficking of lysosomes. Embo J.

[38] Simons C, Dyment D, Bent SJ, Crawford J, D'Hooghe M, Kohlschutter A, Venkateswaran S, Helman G, Poll-The BT, Makowski CC (2017). A recurrent de novo mutation in TMEM106B causes hypomyelinating leukodystrophy. Brain.

[39] Smith KR, Damiano J, Franceschetti S, Carpenter S, Canafoglia L, Morbin M, Rossi G, Pareyson D, Mole SE, Staropoli JF (2012). Strikingly different clinicopathological phenotypes determined by progranulin-mutation dosage. Am J Hum Genet.

[40] Stroobants S, D'Hooge R, Damme M (2021). Aged Tmem106b knockout mice display gait deficits in coincidence with Purkinje cell loss and only limited signs of non-motor dysfunction. Brain Pathol.

[41] Tanaka Y, Chambers JK, Matsuwaki T, Yamanouchi K, Nishihara M (2014). Possible involvement of lysosomal dysfunction in pathological changes of the brain in aged progranulin-deficient mice. Acta Neuropathol Commun.

[42] Tropea TF, Mak J, Guo MH, Xie SX, Suh E, Rick J, Siderowf A, Weintraub D, Grossman M, Irwin D (2019). TMEM106B Effect on cognition in Parkinson disease and frontotemporal dementia. Ann Neurol.

[43] Van Deerlin VM, Sleiman PM, Martinez-Lage M, Chen-Plotkin A, Wang LS, Graff-Radford NR, Dickson DW, Rademakers R, Boeve BF, Grossman M (2010). Common variants at 7p21 are associated with frontotemporal lobar degeneration with TDP-43 inclusions. Nat Genet.

[44] van der Zee J, Van Langenhove T, Kleinberger G, Sleegers K, Engelborghs S, Vandenberghe R, Santens P, van den Broeck M, Joris G, Brys J (2011). TMEM106B is associated with frontotemporal lobar degeneration in a clinically diagnosed patient cohort. Brain.

[45] Werner G, Damme M, Schludi M, Gnorich J, Wind K, Fellerer K, Wefers B, Wurst W, Edbauer D, Brendel M (2020). Loss of TMEM106B potentiates lysosomal and FTLD-like pathology in progranulin-deficient mice. EMBO Rep.

[46] Zhou X, Brooks M, Jiang P, Koga S, Zuberi AR, Baker MC, Parsons TM, Castanedes-Casey M, Phillips V, Librero AL (2020). Loss of Tmem106b exacerbates FTLD pathologies and causes motor deficits in progranulin-deficient mice. EMBO Rep.

[47] Zhou X, Nicholson AM, Ren Y, Brooks M, Jiang P, Zuberi A, Phuoc HN, Perkerson RB, Matchett B, Parsons TM (2020). Loss of TMEM106B leads to myelination deficits: implications for frontotemporal dementia treatment strategies. Brain.

[48] Zhou X, Paushter DH, Feng T, Pardon CM, Mendoza CS, Hu F (2017). Regulation of cathepsin D activity by the FTLD protein progranulin. Acta Neuropathol.

[49] Zhou X, Paushter DH, Pagan MD, Kim D, Nunez Santos M, Lieberman RL, Overkleeft HS, Sun Y, Smolka MB, Hu F (2019). Progranulin deficiency leads to reduced glucocerebrosidase activity. PLoS One.

