# Supplementary material for: Lack of a protective effect of the Tmem106b “protective SNP” in the Grn knockout mouse model for frontotemporal lobar degeneration
Source: Acta Neuropathol Commun. 2023 Jan 27;11:21. doi: 10.1186/s40478-023-01510-3 (PMC9881268; doi:10.1186/s40478-023-01510-3)
Supplement: Supplementary file 1 — Additional file 1: Fig. S1. A Representative images of immunofluorescence stainings of the facial motor nucleus of a wildtype, Tmem106b−/−, Tmem106bT186S/T186S mouse with an antibody against LAMP1(upper panel green, lower panel white). Nuclei are stained with DAPI (upper panel, blue). B Breeding scheme depicting the crossing of Tmem106b+/T186S and Tmem106b−/− to produce Tmem106b−/T186S with one knockout allele and one knockin allele. C Representative images of immunofluorescence stainings of the facial motor nucleus of a Tmem106b−/T186S mouse with an antibody against LAMP1 (upper panel green, lower panel white). Nuclei are stained with DAPI (upper panel, blue). Additional file 1: Fig. S2. Three-dimensional reconstructions of individual Iba1 stained microglia cells of the thalamus of Grn+/+, Grn−/− and Grn−/− T﻿mem106bT186S/T186S mice (pseudocolored, three examples/genotype) used for cell-volume calculation. The panel on the right depicts overview images used for quantification. 50 images were acquired in the Z-direction and assembled with the arivis Vision4D 3.3 software (Zeiss). [file 40478_2023_1510_MOESM1_ESM.pdf]

Supplemental Figure 1

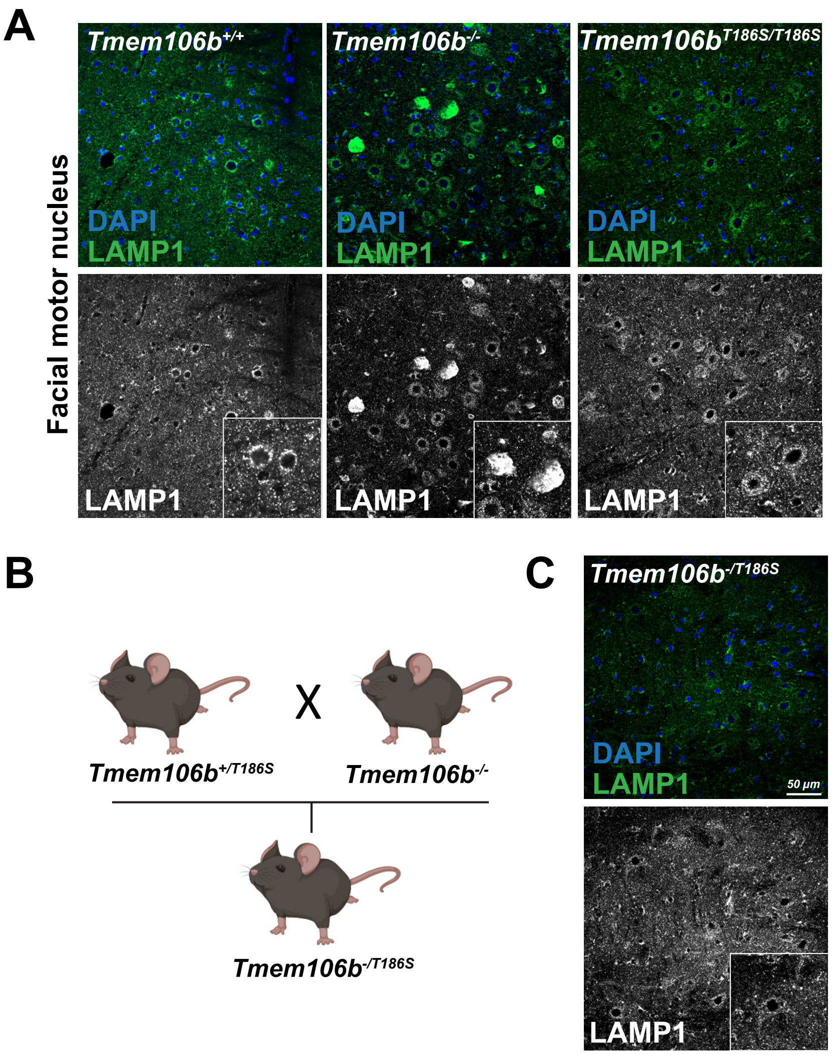

Supplementary Fig.1 (A) Representative images of immunofluorescence stainings of the facial motor nucleus of a *wildtype, Tmem106b*$^{-/-}$, *Tmem106b*$^{T186S/T186S}$ mouse with an antibody against LAMP1 (upper panel green, lower panel white). Nuclei are stained with DAPI (upper panel, blue). **(B)** Breeding scheme depicting the crossing of *Tmem106b*$^{+/T186S}$ and *Tmem106b*$^{-/-}$ to produce *Tmem106b*$^{-/T186S}$ with one knockout allele and one knockin allele. (C) Representative images of immunofluorescence stainings of the facial motor nucleus of a *Tmem106b*$^{-/T186S}$ mouse with an antibody against LAMP1 (upper panel green, lower panel white). Nuclei are stained with DAPI (upper panel, blue).

Supplemental Figure 2

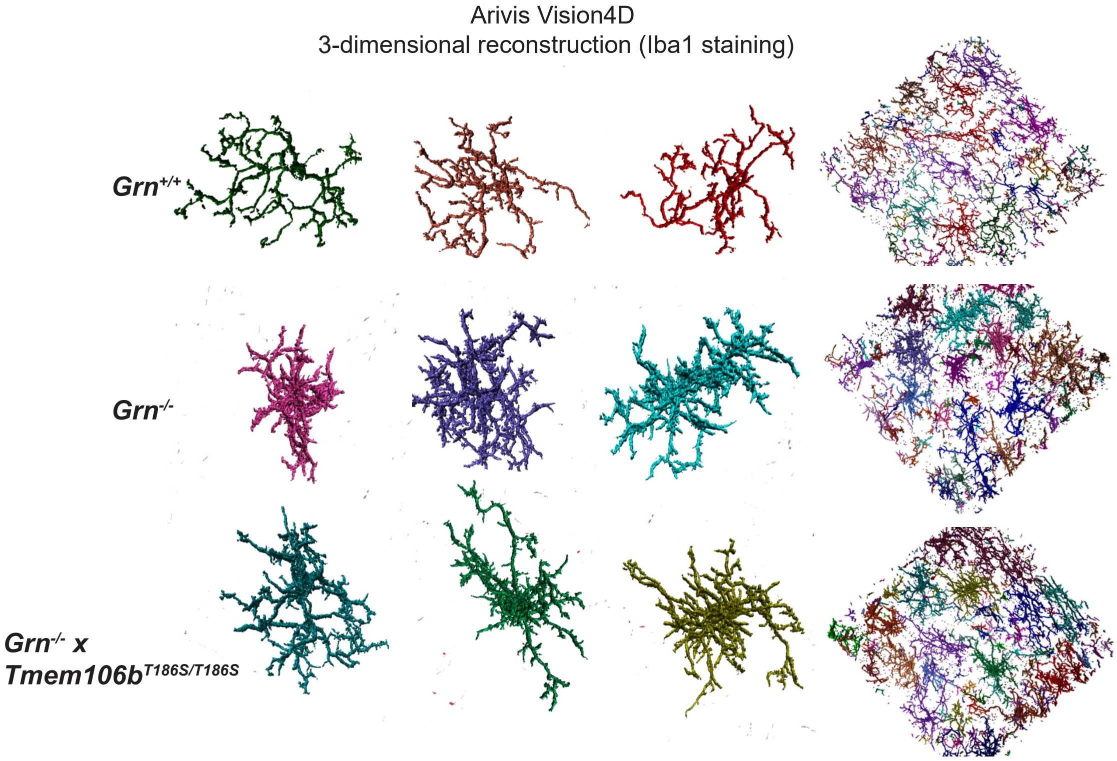

Supplementary Fig.2 Three-dimensional reconstructions of individual Iba-1 stained microglia cells of the thalamus of *Grn*$^{+/+}$, *Grn*$^{-/-}$ and *Grn*$^{-/-}$ *Tmem106b*$^{T186S/T186S}$ mice (pseudocolored, three examples/genotype) used for cell-volume calculation. The panel on the right depicts overview images used for quantification. 50 images were acquired in the Z-direction and assembled with the arivis Vision4D 3.3 software (Zeiss).
